# Trace Level Quantification of 4-Methyl-1-nitrosopiperazin in Rifampicin Capsules by LC-MS/MS

**DOI:** 10.3389/fchem.2022.834124

**Published:** 2022-02-14

**Authors:** Xiaosha Tao, Ye Tian, Wan-Hui Liu, Shangchen Yao, Lihui Yin

**Affiliations:** ^1^ School of Pharmacy, Key Laboratory of Molecular Pharmacology and Drug Evaluation (Yantai University), Ministry of Education, Yantai University, Yantai, China; ^2^ Division of Antibiotics, Institute for Chemical Drug Control, National Institutes for Food and Drug Control, Beijing, China

**Keywords:** rifampicin, nitrosamine impurity, genotoxic impurity, LC-MS/MS, anti-tuberculosis

## Abstract

Rifampicin is a first-line anti-tuberculosis drug. However, in August 2020, the presence of 1-methyl-4-nitrosopiperazine (MNP), a nitrosamine impurity, was detected by the United Stated Food and Drug Administration (US FDA) in rifampicin capsules. Consequently, the development of efficient methods for the detection of MNP is an important objective. In this study, the MNP present in rifampicin capsules was detected using LC-MS/MS. A total of 27 batches from nine manufacturers in the Chinese market were tested, with MNP (0.33–2.36 ppm) being detected in all samples at levels exceeding the maximum acceptable intake limit of 0.16 ppm initially set by the FDA. However, after considering the associated benefits and risks, the FDA-approved limit was revised to 5 ppm; hence, all the samples examined herein exhibited MNP levels well below the required limit. Furthermore, the results of forced degradation experiments suggest that MNP is formed by the thermal degradation of rifampicin.

## Introduction

Genotoxic impurities (GTIs), also referred to as mutagenic impurities, are generally identified using the *Salmonella typhimurium* reverse mutation assay (the Ames test). These impurities are often electrophiles that react with genetic material, resulting in direct or indirect damage to cellular DNA, including the insertion and modification of covalent bonds during DNA alkylation, chromosome breakage, DNA recombination, and DNA replication; this leads to gene mutation and even the onset of cancer (He et al., 2021; Tuesuwan and Vongsutilers, 2021). Compared to pure drugs, the administration of even very small quantities of drugs that contain genotoxic impurities can severely harm patients, especially those who require long-term medication. As such, these drugs must be strictly controlled, with the regulations for genotoxic impurities becoming more encompassing over the past few years; hence, the requirements of drug-regulatory agencies in various countries have become more stringent in relation to genotoxic impurities. In the absence of adequate controls, genotoxic impurities in drugs pose significant clinical risks and compromise patient safety (Committee for Medicinal Products for Human Use, (CHMP), 2006; Müller et al., 2006; Reddy et al., 2015).

*N-*Nitrosodimethylamine (NDMA), a genotoxic impurity, was detected in valsartan in June 2018, while metformin, sartans, ranitidine, and other drugs were later also found to contain genotoxic nitrosamine impurities. Drug contamination by nitrosamine impurities has therefore garnered international attention as a serious drug safety issue (Nowakowska and Pikul, 2012; U.S. Food and Drug Administration, 2019; The European Medicines Agency (EMA), 2020). The International Council for Harmonisation of Technical Requirements for Pharmaceuticals for Human Use (ICH) M7 guideline clearly states that aflatoxins, *N*-nitrosamines, and alkyl-azo compounds are special highly carcinogenic genotoxic impurities; hence, they are grouped together as a cohort of concern (CoC) (ICH, 2020).

Quality control requires impurity trace determination at ppm levels, which can only be addressed by highly sensitive analytical methodologies that pose tremendous challenges to analytical chemists. Liquid chromatography–tandem mass spectrometry has been widely explored for the analysis of genotoxic trace impurities, with such techniques including LC-HRMS and LC-MS/MS (Elder et al., 2008; Nagadeep et al., 2016). The removal of GTIs from (active pharmaceutical ingredients) APIs is of particular importance for the pharmaceutical industry and in the context of API safety assessment. Several processes have therefore been assessed to address this problem, including adequate chemical process design, solid phase extraction, preparative column chromatography, recrystallization and phase exchanges, and fractional distillation (Szekely et al., 2012; Kecili et al., 2013; Schülé et al., 2014).

**FIGURE 1:**
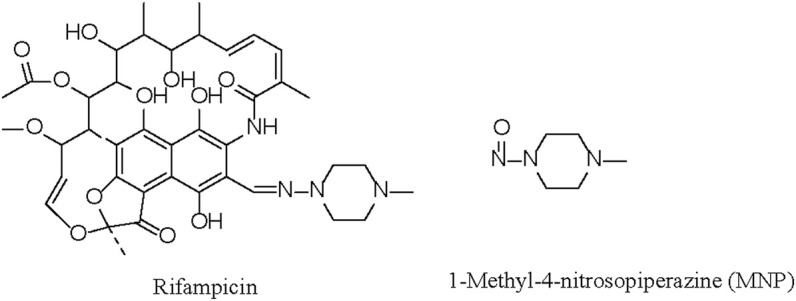
Chemical structures of rifampicin and 1-methyl-4-nitrosopiperazine (MNP).

Rifampicin is a first-line anti-tuberculosis drug, and its structure is shown in Figure 1 (Sensi, 1983). In August 2020, the rifampicin manufactured by Sanofi–Aventis was found by the United Stated Food and Drug Administration (US FDA) to be contaminated with 1-methyl-4-nitrosopiperazine (MNP, Figure 1), a genotoxic nitrosamine impurity. Previously, the FDA had set an acceptable upper limit of 0.16 ppm for the MNP present in rifampicin. However, after considering the benefits and risks of rifampicin for tuberculosis patients, the FDA has temporarily allowed rifampicin containing MNP at levels <5 ppm to be distributed (U.S. Food and Drug Administration, 2020).

Although the FDA has previously published an LC-ESI-HRMS method for the determination of this contaminant, no LC-MS/MS method has been reported. In addition, this FDA method has been reported to adopt a one-point calibration approach, and so led to an overestimation (Wohlfart et al., 2015). Furthermore, we note that the LC-ESI-HRMS and LC-MS/MS techniques can complement one another. Thus, to provide an improved understanding of the contents of relevant products in China and to provide technical support for future regulatory actions, we herein report the development of an HPLC-MS/MS-based method for the detection of MNP in rifampicin capsules, and subsequently determine the MNP contents of rifampicin capsules approved for use in China. Furthermore, forced degradation experiments are employed to determine the source of the MNP contaminant present in rifampicin.

## Materials and Methods

### Materials and Instruments

Rifampicin capsules (a total of 27 sample batches) were purchased from nine manufacturers on the Chinese market. Ammonium formate, formic acid, and LC-MS grade methanol were purchased from ThermoFisher Scientific Inc. (United States). An MNP reference sample (96.3%, batch number: 1119-RB-1010) was purchased from CATO Research Chemicals Inc. (United States).

The instruments used in the study included a Shimadzu LC-20AD liquid chromatograph (Japan) fitted with a photo-diode array (PDA) detector and a LabSolution workstation, an AB SCIEX 6500 Qtrap mass spectrometer (United States), a Shimadzu Shiseido Nanospace HPLC system (Japan) with an Analyst workstation, a Mettler Toledo XP205 electronic balance (Switzerland), and a ThermoScientific MAXQ 6000 shaker (United States).

### Sample Preparation

Methanol was used as the blank solution. For sample preparation, MNP (10 mg) was dissolved in MeOH (100.0 ml) to reach a concentration of 100 μg/ml. An aliquot (100 µl) of this stock solution was diluted with MeOH to 50.0 ml in a volumetric flask to obtain a stock standard preparation (SSP) with a concentration of 200 ng/ml. The working standard preparation (WSP) with a concentration of 2 ng/ml was prepared by dilution of the SSP (250 μl) with MeOH to 25.0 ml in a volumetric flask.

The solutions employed to determine the limit of detection (LOD) and the limit of quantitation (LOQ) were prepared by dilution of the WSP to give concentrations of 0.1 and 0.2 ng/ml, respectively.

Seven solutions (0.2, 1, 5, 10, 25, 100, and 200 ng/ml) were prepared from the SSP and stock solutions to cover the range of MNP concentrations detected in the samples (i.e., 0.2–200 ng/ml).

The rifampicin capsules were tested by sampling a single capsule content (∼150 mg of rifampicin) and placing it in a 50 ml centrifuge tube, to which methanol (10 ml) was added, and the mixture was shaken well for 10 min. The supernatant was then collected and filtered, and the filtrate was saved for subsequent testing.

The SSP was added to the contents of a rifampicin samples to prepare the recovery test solutions, such that spiking was achieved to three final concentrations, namely low (5 ng/ml), medium (10 ng/ml), and high (25 ng/ml) concentrations. Three replicates were prepared for each concentration, which served as the recovery test solutions.

The stability of the rifampicin capsule test samples to heat and a high humidity were examined under storage in constant-temperature humidity boxes at 25°C (75% RH, ultraviolet lighting), 25°C (75% RH), 40°C (75% RH), and 60°C (75% RH), and the MNP contents of these samples stored under the above conditions for 3, 7, 10, and 30 days were measured.

For other forced degradation studies, the samples were subjected to stress conditions according to ICH guidelines. For the acidic and alkaline degradation experiments, a sample of rifampicin (150 mg) was dissolved in a 0.1 M HCl or NaOH solution (2.0 ml) and maintained at room temperature for 3 h. Prior to further dilution with methanol, these samples were neutralized.

### LC-MS Conditions

#### LC Conditions

An ACE UltraCore Super C18 (4.6 × 50 mm, 2.5 μm; Agilent, United States) chromatography column (stationary phase: octadecyl silane chemically bonded to silica gel) was used for LC analysis. An aqueous 1 mM ammonium formate solution was used as mobile phase A and methanol was used as mobile phase B; the gradient elution conditions listed in Table 1 were applied at a column temperature of 35°C using an injection volume of 3 μl.

**TABLE 1:** LC gradient elution conditions.

Time (min)	Mobile phase A (%)	Mobile phase B (%)	Flow rate (ml/min)
0.0	60	40	0.5
2.0	60	40	0.5
6.0	0	100	0.5
6.1	0	100	0.8
12.0	0	100	0.8
12.1	60	40	0.5
17.0	60	40	0.5

Note: MNP exhibited a retention time of approximately 1.26 min.

#### MS Conditions

The samples were analyzed using the abovementioned triple quadrupole tandem mass spectrometer under positive-mode ESI conditions with multiple reaction monitoring (MRM). The values of the various parameters were as follows: curtain gas pressure: 25.0 psi; collision gas pressure: 8 psi; ion spray (IS) voltage: 4,000 V; TEM (drying gas temperature): 450°C; ion source gas 1 (spraying gas) pressure: 65 psi; ion source gas 2 (drying gas) pressure: 45 psi; polarity: positive; MS monitoring time: 0.5–3.5 min to MS and 3.5–17.0 min to waste. The MRM ion pair values of the parameters employed are listed in Table 2.

**TABLE 2:** MRM ion pair for 1-methyl-4-nitrosopiperazine (MNP).

Parent ion (m/z)	Daughter ion (m/z)	Declustering potential (DP, V)	Collision energy (CE, V)	Polarity
130.1	100.1^a^	48	12	Positive
	58.1	48	22	Positive

a Quantitative ion.

## Results and Discussion

### Verifying the Methodology

An aliquot (3 μl) of the blank solvent and the rifampicin capsule test solution were injected into the LC-MS system and the corresponding chromatograms were recorded. The results show that peaks corresponding to the blank solvent, to rifampicin, and to any other related substances do not interfere with the MNP peak. Subsequently, aliquots (3 μl) of the LOQ (0.2 ng/ml) and LOD (0.1 ng/ml) solutions were injected, which revealed signal-to-noise ratios (S/Ns) of 48.5 for the MNP peak in the LOQ solution and 10.2 for the MNP peak in the LOD solution, which meet the detection sensitivity requirements.

For evaluation of intra-day precision, six replicate sample solutions (10 ng/ml) prepared using the reference solution were analyzed on the same day. For the inter-day precision, two duplicate sample solutions (10 ng/ml) prepared using the reference solution were analyzed for three consecutive days. The RSDs of the obtained peak areas were calculated to be 2.27 and 7.27%, respectively, which were indicative of a good method repeatability. A standard curve was generated by plotting the solution concentration (Y) against the peak area (X) using 3 μl aliquots of the 0.2–200 ng/ml MNP reference solutions; linear regression analyses revealed a good linearity in the 0.2–200 ng/ml concentration range (*r* = 0.9999).

The recovery test solution was added to the capsule solution to give three final spiking concentrations, namely low (5 ng/ml), medium (10 ng/ml), and high (25 ng/ml) concentrations, with three replicates prepared at each concentration. The LC-MS results showed an average recovery rate of 87.05% for all three concentrations (Table 3).

**TABLE 3:** Spike recovery rates (%) of the rifampicin capsules (n = 9).

No	Low (5 ng/ml) concentration	M (%)edium (10 ng/ml) concentration	H (%)igh (25 ng/ml) concentration
1 (%)	87.86	84.66	87.44
2	88.53	86.82	86.99
3	82.75	90.05	88.33
Average	86.38	87.18	87.59
RSD (%)	3.66	3.11	0.78
Average spike recovery rate = 87.05%			
Average RSD = 2.52%			

The above data therefore demonstrate that the developed method exhibits a high linearity and accuracy, as well as good LOD and LOQ values, thereby indicating that it can effectively detect the presence of MNP in rifampicin capsules.

### Detecting MNP in Rifampicin Batches

**FIGURE 2:**
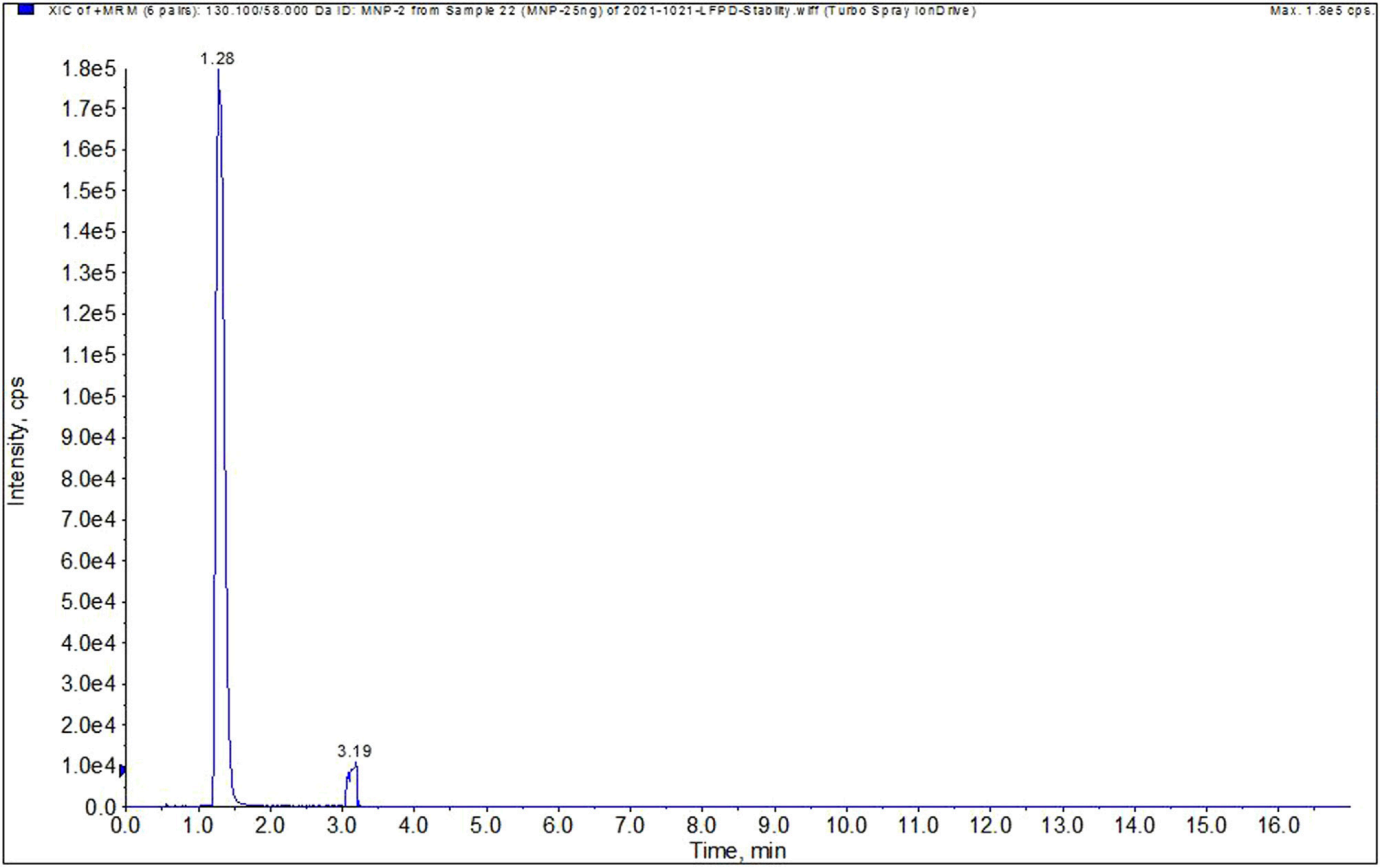
Typical MRM chromatogram of MNP.

In this study, the retention time of MNP was 1.28 min (Figure 2) and that of rifampicin was 6.7 min. To prevent a high concentration rifampicin entering and polluting the MS ion source, it is necessary to prolong the elution time to completely elute rifampicin from the column. If this is not carried out, ion inhibition against the trace MNP content will take place. It has been reported that the LC-HRMS method published by the FDA adopted a one-point calibration approach, which led to overestimation of the MNP content. Thus, our LC-MS/MS method (i.e., triple quadrupole mass spectrometer, MRM mode) was applied for the quantitative determination of the trace quantities of MNP present in rifampicin. The LOD and LOQ values for MNP were determined to be 0.0067 and 0.013 ppm, respectively, which are superior to the values of 0.01 and 0.017 ppm achieved using the FDA method.

**FIGURE 3:**
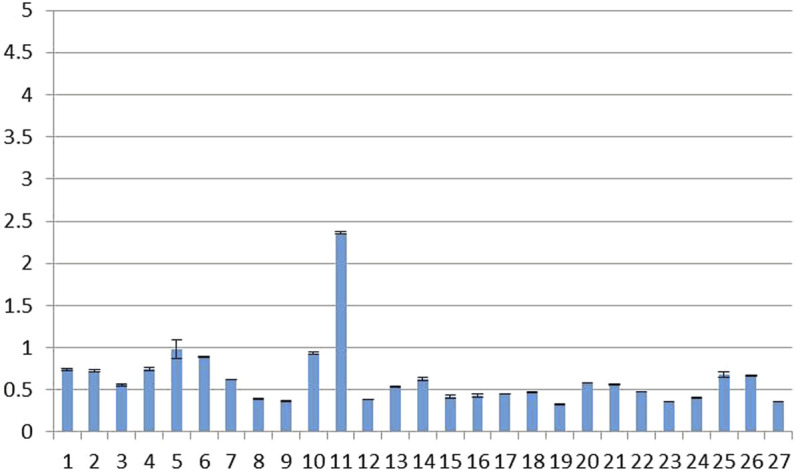
MNP contents of 27 batches of rifampicin capsules (temporarily threshold concentration: 5 ppm).

As shown in Figure 3, the MNP contents of the 27 batches of rifampicin capsules sampled in this study were found to be within the range of 0.33–2.36 ppm; this is 2–15 times higher than the original acceptable limit of 0.16 ppm, but below the FDA-revised limit of 5 ppm, which was established after comprehensively considering the benefits and risks associated with the use of rifampicin for tuberculosis patients.

**FIGURE 4:**
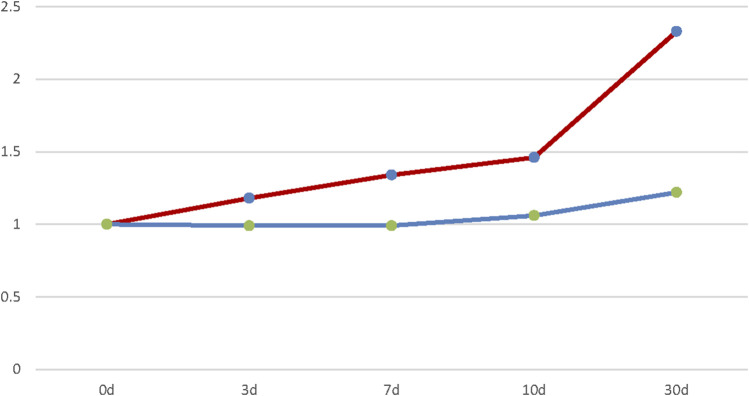
MNP contents of rifampicin capsules: red: 60°C, 75% RH; blue: 40°C, 75% RH.

A variety of factors can lead to chemical contamination with trace amounts of genotoxic MNP impurities, the main sources of which can be traced back to contaminated raw materials, solvents, and catalysts, which are employed in the production processes (Szekely et al., 2015). Forced degradation experiments conducted under acidic, alkaline, UV-irradiation, high-humidity, and high-temperature conditions revealed significantly higher MNP contents in rifampicin when heated (Figure 4). For example, the MNP content increased by 25% following storage at 40°C for 30 days, and it more than doubled when stored at 60°C for 30 days. This suggests that MNP is a thermal degradation product of rifampicin. In contrast, no significant increase in the MNP content was observed in rifampicin when exposed to acidic, alkaline, UV irradiation, and high-humidity conditions. These results further suggest that MNP is likely produced by the thermal degradation of rifampicin; therefore, we conclude that the temperatures used during production and sample storage should be strictly controlled.

## Conclusion

We herein reported the development of an LC-MS/MS-based method for detecting MNP, a genotoxic nitrosamine impurity. This method was fully validated and presents a good linearity, specificity, accuracy, and precision. Using this method, the LOD and LOQ values for MNP were 0.0067 and 0.013 ppm, respectively. Subsequently, the MNP levels in 27 batches of rifampicin samples obtained from different manufacturers in China were determined, and it was found that all samples exhibited MNP levels within the revised FDA-approved upper limit of 5 ppm. This study therefore comprehensively reflects the level of MNP in rifampicin from the Chinese market, and the results presented indicate that the formation of MNP is directly related to temperature, and so its levels may be controlled through careful temperature regulation during manufacture and storage. Further studies are therefore required to determine the corresponding degradation mechanism.

## Data Availability

The original contributions presented in the study are included in the article/supplementary material, further inquiries can be directed to the corresponding authors.
